# Putative Pacemakers in the Eyestalk and Brain of the Crayfish *Procambarus clarkii* Show Circadian Oscillations in Levels of mRNA for Crustacean Hyperglycemic Hormone

**DOI:** 10.1371/journal.pone.0083937

**Published:** 2013-12-31

**Authors:** Janikua Nelson-Mora, Julio Prieto-Sagredo, Rosaura Loredo-Ranjel, María Luisa Fanjul-Moles

**Affiliations:** Laboratorio de Neurofisiología Comparada, Departamento de Ecología y Recursos Naturales, Facultad de Ciencias, Universidad Nacional Autónoma de México, Distrito Federal, México; Federal University of Rio de Janeiro, Brazil

## Abstract

Crustacean hyperglycemic hormone (CHH) synthesizing cells in the optic lobe, one of the pacemakers of the circadian system, have been shown to be present in crayfish. However, the presence of CHH in the central brain, another putative pacemaker of the multi-oscillatory circadian system, of this decapod and its circadian transcription in the optic lobe and brain have yet to be explored. Therefore, using qualitative and quantitative PCR, we isolated and cloned a CHH mRNA fragment from two putative pacemakers of the multi-oscillatory circadian system of *Procambarus clarkii*, the optic lobe and the central brain. This CHH transcript synchronized to daily light-dark cycles and oscillated under dark, constant conditions demonstrating statistically significant daily and circadian rhythms in both structures. Furthermore, to investigate the presence of the peptide in the central brain of this decapod, we used immunohistochemical methods. Confocal microscopy revealed the presence of CHH-IR in fibers and cells of the protocerebral and tritocerebal clusters and neuropiles, particularly in some neurons located in clusters 6, 14, 15 and 17. The presence of CHH positive neurons in structures of *P. clarkii* where clock proteins have been reported suggests a relationship between the circadian clockwork and CHH. This work provides new insights into the circadian regulation of CHH, a pleiotropic hormone that regulates many physiological processes such as glucose metabolism and osmoregulatory responses to stress.

## Introduction

The metabolic crustacean hyperglycemic hormone (CHH) is produced by neuroendocrine cells of the X-organ sinus gland (XO-SG) complex in the eyestalks of decapod crustaceans. This hormone is involved in regulating the glucose levels in the haemolymph, and the participation of CHH in other physiological processes such as osmoregulatory responses to stress has been well established [1]. Due to the antigenicity of CHH, polyclonal antibodies against purified CHH from several species can be easily produced, allowing the immunofluorescent, cytochemical and morphological identification of the CHH-producing cell system in numerous crustaceans (for review, see [2]). During the last decades, data obtained from bioassays, immunocytochemistry and morphometry have provided information about the physiology of the CHH-producing cells in crayfish; this information includes the synthesis, storage and release of the hyperglycemic hormone. These approaches have demonstrated the diurnal rhythm of the system: as CHH is released at the onset of a dark period, synthetic activity increases 2 h before the liberation of CHH [3], [4], [5]. The regulation of CHH synthesis and its release occurs through synaptic input to the CHH-axon ramifications in the medulla terminalis. Biogenic amines, especially serotonin, have been postulated to be involved in the regulation of CHH release [6], [1]. Although the XO–SG is considered the main locus of neuropeptide production, CHH/MIH peptides have been detected at other sites in crustacean organs. Small amounts of CHH have been detected by radioimmunoassay in the pericardial organs of *Carcinus maenas* [7]. Other authors isolated two cDNA sequences encoding two different CHH preprohormones from the sinus gland of *Homarus americanus* and showed that both the sinus gland and the ventral cord express the same *CHH* mRNA [8]. Recently, CHH has been detected by immunocytochemistry in other regions of the nervous system of decapods,such as the subesophageal ganglion of *Homarus* [9] neurons in the so-called second thoracic roots of the ventral nerve cord in lobsters [10], the retina of the crayfish *Procambarus clarkii* (*P. clarkii*) [11] and the brain of other non-decapod crustacean species [12], [13], [14]. Nussbaum and Dircksen [15] reported eight CHH-immunoreactive perikarya in the brain of the isopod *Oniscus asellus* [15] *;* furthermore, the neurosecretory pathway leading to the SG has been described in detail, and collaterals that formed dendritic branching were detected in the central protocerebrum. Those authors also identified eight CHH-immunoreactive cells in *Porcellio scaber* and *Ligia oceanica*. Azzouna et al. [14] who worked with *A. vulgare* brains, reported six immunoreactive cells from a previously described sub-population of β-cells [13] in the medial and anterior parts of the brain. However, until now, the presence of either the CHH hormone or its transcript in the supraesophageal ganglia (brain, *i.e* median brain or median protecerebrum) of decapods, [16] particularly crayfish, has not been demonstrated.

Although the structure and biochemical properties of CHH have been extensively studied and CHH has been isolated and sequenced, the total number of neuropeptides that belong to the CHH subtype and its gene organization, expression pattern and evolutionary relationships between its peptides have not been completely resolved. Furthermore, the total number of CHH genes has not been determined for any species. Approximately 40 genes have been reported for different species of decapods, including shrimp, crab, crayfish and lobster [6], [1] There are two variants of CHH (I and II) in *P. clarkii*, and these forms can be separated by reversed-phase high-performance liquid chromatography [17]. Both variants contain 72 amino acid residues with three disulfide linkages at positions 7–43, 23–39 and 26–52, and the variants differ from each other by the D/L epimerization of phenylalanine at position 3. These sequence differences are important because they result in variations in interspecific hyperglycemic activity. Different isoforms in crayfish have different hyperglycemic effects [18], [19], [20], [21], [22].

As mentioned above, some studies have shown that the synthesis, secretion and levels of CHH in the eyestalk and haemolymph are controlled by an endogenous biological clock in various species of crayfish [3], [4], [5], [23], [24]. To the best of our knowledge, however, the circadian control of *CHH* transcription has not been demonstrated. A non-radioactive *in situ* hybridization procedure for localizing the mRNA that encodes the CHH in the eyestalk of the crayfish *Orconectes limosus* was developed in 1991 [25]. The circadian control of *CHH* transcription has not been confirmed despite the importance of this information for full comprehension of the temporal control of CHH synthesis and its post-transcriptional modifications. Therefore, in this study, we contributed to the knowledge of the circadian control of *CHH* transcription and localization in two ways. First, we isolated and cloned a similar *CHH* mRNA fragment from two of the putative pacemakers of crayfish, the eyestalk and the brain, and investigated whether the levels of this transcript showed circadian variations. The levels of this mRNA oscillate, showing daily and circadian variations in both structures. Second, using immunochemical methods, we searched for CHH in the cell body clusters and neuropils in the brain of *P. clarkii*. Interestingly we located CHH-positive neurons in the brain of *P. clarkii*, thereby demonstrating the presence of this neuropeptide in decapod brains for the first time.

## Materials and Methods

### Ethical Statement

All experiments complied with the current laws of Mexico, the country in which they were conducted. No specific permits were required for the studies that did not involve endangered or protected species. Individuals were maintained under appropriate laboratory conditions to guarantee their welfare and responsiveness. After the experiments were completed, crayfish were sacrificed by hypothermia and decapitation.

### Animals and Experimental Design

The *P. clarkii* crayfish used for this study were collected from streams near the Conchos River, Chihuahua, Mexico. The animals were acclimatized to the lab for two weeks prior to being used in the experiments. Aquaria were maintained with 12∶12 h light: dark cycles (LD) and 5.9 mg/l O_2_ and were kept at 22°C. Crayfish were fed three times per week with shrimp pellets (Nestlé Purina, St. Louis, MI, U.S.A.) and vegetables. After this period, 82 animals were placed under the following experimental conditions for two weeks. Ten animals were maintained in an LD cycle of 12∶12 h with lights on at 07∶00 h. Total RNA was isolated from these animals and was used for sequencing and the construction of calibration curves. A second experimental batch of 36 crayfish was divided into two groups. Half of the animals were kept in an LD cycle of 12∶12 h with lights on at 07∶00 h, and the other half were kept in a DL cycle of 12∶12 h with lights on at 20∶00 h. At the end of the experimental period, the animals were sacrificed, and tissue samples from the brain and optic lobe were collected every 4 h beginning at 08∶00 h; in total, samples were collected at six time points during the day. The final group included 36 crayfish, and these animals were placed in the same light-dark and dark-light conditions as the second group. However, after a two week period, the lights were shut off, and the animals were subjected to darkness for three days. On the fourth day of darkness, the crayfish were sacrificed, and tissue samples from the brain and optic lobe were collected at same time points as described above. The temporal expression of mRNA was studied in the second and third batches of experimental animals.

### Molecular Determination of CHH cDNA

#### Tissue collection and RNA isolation

All the molecular biology protocols are in compliance with the MIQE guidelines (Minimum information for publication of quantitative Real-time PCR experiments [26]). Six animals were sacrificed by decapitation at each sampling time. The night samples were collected from the DL 12∶12 h group of animals. To collect the samples at the correct time point, the LD animals were sacrificed first, and then the DL animals were sacrificed. In these groups, the light changes were delayed by one hour to collect the samples at the correct time point. The brain of each animal was dissected and immediately placed in a sterile 1.5 ml microcentrifuge tube containing 300 µl of cold Tripure (F. Hoffmann-La Roche AG, Basel, Switzerland) solution. The sample was homogenized using a tissue grinder with a sterile pestle and by vortexing. The optic lobe was dissected in cold RNAlater solution (Ambion, Life technologies Corp., Carlsbad, CA, U.S.A.) to preserve the total RNA and was placed immediately in cold Tripure (Roche) solution for homogenization, as performed for the brain samples. Total RNA isolation was performed using Tripure (Roche) according to the manufacturer’s instructions and the Chomczynski and Sacchi method [27]. Briefly, after tissue homogenization, chloroform was added to each sample to isolate the RNA from the DNA and protein. The aqueous phase was placed in a separate sterile tube, and 100% isopropanol (Amresco Inc., Solon, OH, U.S.A.) was added to precipitate the RNA. After centrifugation, the pellet was washed with cold 75% ethanol and centrifuged. After discarding the ethanol, the pellet was allowed to air dry in an isolated chamber to avoid contamination. The total RNA pellet from the brain and eyestalk was hydrated with 6 µl of sterile water. Once hydrated, the total RNA was treated with DNase using the DNase turbo kit (Ambion) according to the manufacturer’s instructions. After hydration, the total RNA was placed at −20°C. The RNA sample was thawed once within 2 d of collection when it was used to create cDNA. An aliquot of each sample was used to quantify and verify the quality of the total RNA using a NanoDrop 100 spectrophotometer (Thermo Fisher Scientific, Waltham, MA, U.S.A.).

#### RT-PCR, cloning and sequencing (end point PCRs)

Based on the sequence analysis reported by Chen et al. [28], a pair of oligonucleotides was designed to amplify the highly conserved regions within the coding region of the CHH transcript. These primers were designed using the GenBank sequence (AB0027291.1) and the Oligo (vs. 7, Molecular Biology Insights Inc., Cascade, CO, U.S.A.) software. Table 1 shows the primer sequences and the expected product length for the quantitative PCR (qPCR) experiments. All oligonucleotides were synthesized by IDT (Integrated DNA Technologies Inc., Coralville, Iowa, U.S.A.).

**Table 1:** Oligonucleotide sequences.

Gene	Oligonucleotide	Sequence*
CHH	CHHup	5′-GCTTGACCGAGTGTGTGAAGATT-3′
	CHHdwn	5′-CAAGAGAAGGTCGTCAAGGCATT-3′

Product length: 120 bp.

Total RNA obtained from the first experimental batch was used in reverse transcriptase PCR (RT-PCR) to produce cDNA. The reactions were prepared by mixing 0.5 µg of random hexamers, 2 µg of total RNA and 2 µl of nuclease free water in each reaction tube. The tubes were heated to 70°C for 5 min and then chilled on ice for 2 min. After a brief spin, 5 µl of the reaction buffer (MgCl_2_ final concentration 3 mM; Promega, Corp., Madison, Wisconsin) was added to the reaction; together with a nucleotide mix (final concentration 0.5 mM each nucleotide; Altaenzyme, Alberta, Canada), 20 units of Recombinant RNasin ribonuclease inhibitor (Promega), 200 units of M-MLV reverse transcriptase (Promega) and 8 µl of nuclease-free water. The tubes were then incubated for 60 min at 42°C in a Techne TC312 thermal cycler (Techne, Bibby Scientific Ltd., Staffordshire, UK). Reverse transcriptase was inactivated after the extension phase by incubation at 70°C for 15 min.

Amplification of the mRNA fragments was performed using a reaction mix composed of 1 µl of 10x buffer (Altaenzymes), dNTP mix (2 mM final concentration of each nucleotide, Altaenzymes), 2.3 mM MgCl_2_, 500 nM of each primer, 0.1 units of recombinant Taq DNA polymerase (Altaenzymes) and nuclease-free water up to 10 µl. A total of 2 µl of the synthesized cDNA was added to the mix, and after gentle mixing, the reaction was performed using the following conditions in a Techne thermal cycler (Techne): initial denaturation at 94°C for 2 min, followed by 35 cycles of denaturation for 1 min at 94°C, annealing for 45 s at 60°C and extension for 1 min at 72°C. A final 10 min extension period was then performed at 72°C.

PCR products were cloned and sequenced at the Proteogenomic Unit, Neurobiology Institute, Universidad Nacional Autónoma de México, (Juriquilla, Querétaro, México), using the pGEMT-EASY vector (Promega). After verifying that the cloned product was present in the positive colonies, the product was sequenced using BigDye Terminator v.3.1 Cycle sequency kit (Applied Biosystems Inc., Carlsbad, CA, U.S.A. ) in an ABI PRISM 310 Genetic Analyzer (Abi Prism, Life Technologies Corp. Carlsbad, CA, U.S.A.).

**Figure 1:**
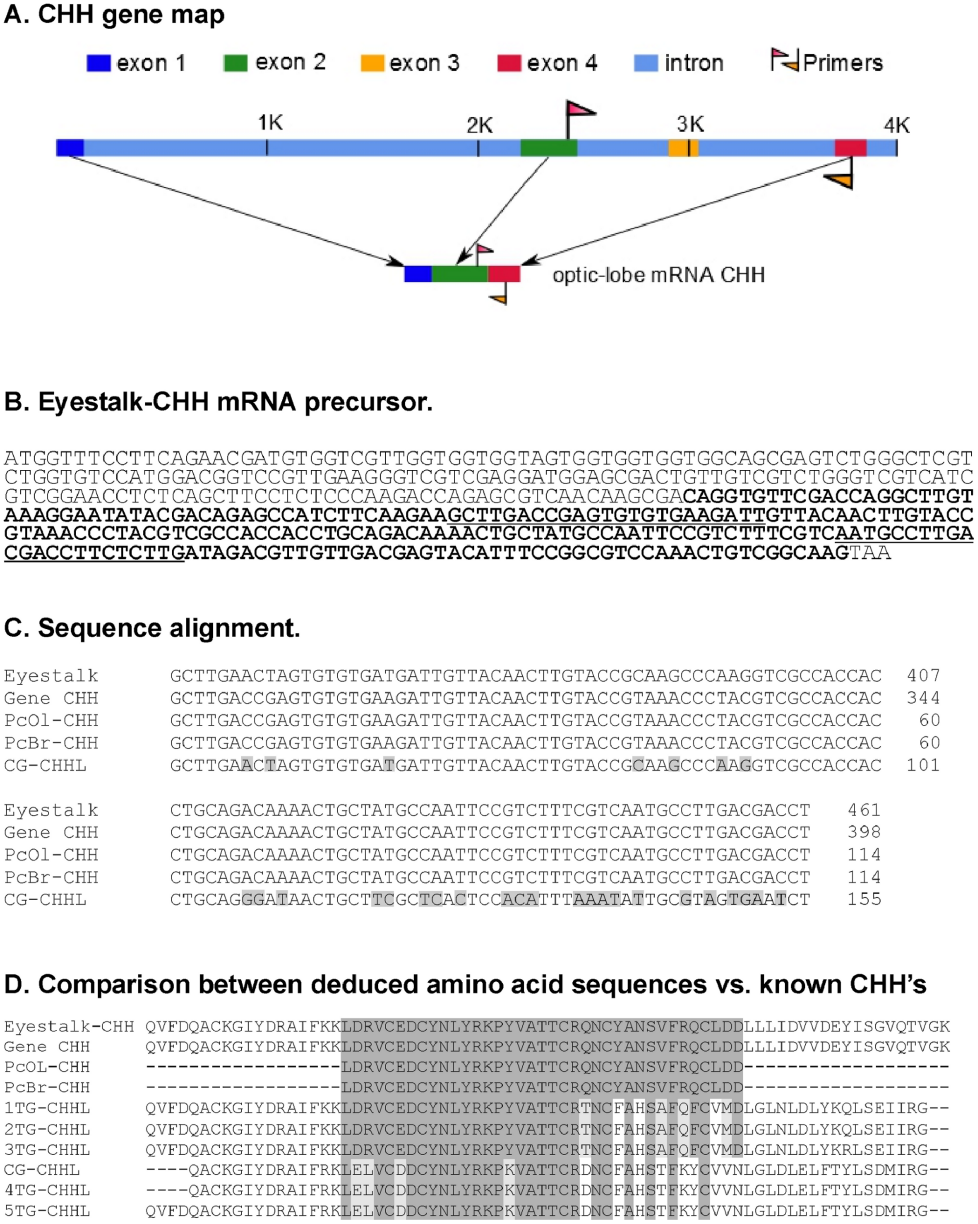
CHH gene map based on GenBank sequence AF474409. **1.** The position of the exons, introns and the PCR primers within the sequence are marked. The expected amplicon expands between exon 2 and 4. B. The primer position is shown (underlined base pairs) over the eyestalk CHH mRNA precursor sequence (Gene Bank acc. no. AB027291.1). Bold letters represent the coding region of the CHH protein. C. Base pair sequence alignment between the eyestalk CHH mRNA precursor (Eyestalk-CHH) and the amplicon sequences obtained in this work: the optic lobe sequence, PcOL-CHH, the brain sequence, PcB-CHH and the brain CHH-like sequence, CG-CHHL (GenBank acc. no. AY256877.1). D. The amino acid sequence alignments between Eyestalk-CHH, the deduced amino acid sequence from the optic lobe and brain amplicons obtained in this work, PcOL-CHH and PcB-CHH, the CHH-like sequence from the thoracic ganglia (1–5 TG-CHHLs; GenBank acc. nos. AAL79193.1, AF474408.1, JF311403, AY256876.1, JX856149) and the deduced amino acid sequence from CG-CHHL.

ClustalW (v 2.1) software [29] was used to align the obtained sequences against sequences acquired from GenBank for each gene. Fig. 1a,b shows the positions of the oligonucleotides in the *CHH* mRNA sequence (GenBank acc. nos. AB027291.1 and AF474409.1). This figure also shows the alignment and the percent identity of the obtained amplicons relative to the reference sequence as well as other CHH-like sequences reported for *P. clarkii* (Fig. 1c). Fig. 1d shows the deduced amino acid sequences of the amplicons obtained in this work and the comparison of the *P. clarkii* eyestalk CHH sequence with previously reported CHH-like sequences from the thoracic ganglia and cerebral ganglion reported for this species.

### Quantitative PCR (qPCR)

#### qPCR oligonucleotides

The same *CHH* oligonucleotides that were used for the RT-PCR analysis were also used for the qPCR assays. The resulting amplicon encompassed exons 2 and 4 of the *CHH* gene (GenBank accession no. AF474409.1); because this amplicon spanned the splice junction, it was possible to distinguish between samples contaminated with genomic DNA and uncontaminated samples.

The optimal primer concentrations for qPCR were determined using a primer matrix experiment in which reactions were prepared without cDNA using a combination of primers at concentrations ranging from 100 to 300 nM of each primer. Secondary structures were not produced at any of the tested concentrations of the forward and reverse primers, as evaluated by melting curves. Therefore, we used a concentration of 200 nM for each primer in the qPCR experiments.

#### Real time PCR (qPCR) reaction conditions and validation

The qPCR reaction was prepared manually using a SYBR green mix (Kapa SYBR Fast qPCR kit (2x) universal, Kapa Biosystems, Inc., Woburn, MA, U.S.A.). In brief, we used 200 µl PCR tubes placed in cold metal racks, and to each tube, we added 10 µl of SYBR FAST mix (Kapa Biosystems), 200 nM each primer, 7.4 µl of sterile, nuclease-free water and 1 µl of cDNA. The final MgCl_2_ concentration in the mix was 2.5 mM. Each sample was analyzed in triplicate. Once the reaction mixture was ready, the tubes were placed in a Rotor GeneQ Thermal cycler (Qiagen GmbH, Hilden, Germany), and the following reaction conditions were used: enzyme activation for 3 min at 95°C, followed by 40 cycles of denaturation for 10 s at 95°C and annealing and extension for 20 s at 60°C. At the end of the amplification protocol, a melting curve analysis was performed to verify the melting pattern of the product and to determine if there was any sample contamination. The melting temperature range was between 50 and 95°C.

To calculate the efficiency of the reaction, calibration curves were performed using cDNA templates created from the optic lobes samples. The PCR product of the CHH amplicon was purified from a high-concentration PCR reaction. We then calculated the volume containing 30×10^6^ copies per reaction tube of the product; using this stock, 1∶10 serial dilutions were used to produce a standard curve down to 300 copies/tube. The reaction efficiency in this range was 1.01 with an R^2^ value of 0.996, an intercept of 35.109 and a slope of −3.294. The limit of detection was 300 copies/tube with a variation of 1.4%.

#### qPCR analysis

All calculations were performed using Rotor-Gene Q-Pure Detection software (v 1.7, Qiagen). The regression analysis of the calibration curves was confirmed using PASW statistics (vs. 18.0.0, IBM Corp., Armonk, NY, U.S.A.). The Cq was determined using the automated tool in the qPCR software. If a variation of greater than 5% was observed between the triplicates of each sample (calculated based on the number of copies/tube, not the Cq), the data were rejected, and the samples were processed again. The non-template controls (negative) did not show any amplification, and therefore, it was not possible to calculate a valid Cq for these reactions.

The mRNA expression levels of the 18S rRNA and G3PDH genes were also analyzed; nevertheless, they presented statistically significant oscillations in several of our experimental conditions (data not shown) and were not used for normalization. We then decided to perform absolute quantification of our data instead of relative quantification [30], [31].

### Statistical Analysis

To identify significant oscillations (*P<*0.05) in the mRNA expression, we used cosinor analysis [24], and one way ANOVA with LSD post hoc comparisons.

### Histology

For immunocytochemical analyses, neural tissues from six organisms were fixed in 4% paraformaldehyde at 4°C for 24 h. After the samples were washed with PBS (0.1 M sodium phosphate, pH 7.4, 0.45 M NaCl, and 0.4% Triton X-100), they were placed in 20% then 30% sucrose solution at 4°C for 24 h each. Subsequently, the tissues were included in Tissue Tek’s O.C.T compound (Sakura Finetek USA Inc., Torrance, CA, U.S.A.) at 20°C and processed using Anglia Scientific 620 cryostat (Anglia Scientific Ltd, London, U.K.) at −20°C. The 10 µm sections were collected and washed with PBS (pH 7.4) for 15 min and then incubated at 4°C for 48 h in a blocking solution containing 2% bovine serum albumin (BSA), 5% milk and 0.1% Triton X-100. After several washes with PBS, a primary rabbit antiserum raised against Norway lobster CHH (1∶250) was diluted in a modified blocking solution (2% BSA, 1% milk and 0.1% Triton X-100) and was added to the tissues. The samples were then incubated for 2 h at room temperature followed by incubation at 4°C overnight. The antibody was previously characterized by [32] and was kindly provided by P. Giulianini. The tissues were then incubated with a secondary antibody (DyLight 594 Goat anti-rabbit, Genetex, San Antonio, TX, U.S.A.) at a 1∶750 dilution for 2 h at room temperature. The nuclei were stained with DAPI (Dako Inc., Glostrup, Denmark) (1∶1000) for 10 min. Finally, the tissues were prepared for confocal laser scanning microscopy. The specificity of the immunoreaction was tested using two methods: (i) the control sections were treated in the same manner as the experimental samples, except that the primary antiserum was omitted; and (ii) the antibody was preabsorbed with a crude extract of the *P. clarkii* sinus gland prior to incubation of the sections as previously described [24].

### Assessment of Light Parameters

The photoperiod was provided by neon lamps that were turned on and off at 07∶00 and 19∶00 h, respectively, by a timer. The light quantum scalar irradiance was calibrated using a photoradiometer equipped with a spherical submarine sensor (LiCor models LI-189 and LI-193SA; LiCor, Lincoln, Nebraska, U.S.A). Intensity values were set to the lowest light irradiance values that crayfish are exposed to in their natural environment.

## Results and Discussion

### RNA Sequence Determination

Two oligonucleotide primers (CHH forward and reverse; Table 1) were designed based on the *CHH* mRNA sequence reported by Yasuda-Kamatani and Yasuda (GenBank acc. no. AB027291.1). A 114-bp fragment was amplified, cloned and sequenced from the optic lobe. The obtained sequence showed 100% sequence identity with a previously reported complete sequence of *P. clarkii CHH* mRNA (GenBank acc. no. AB027291.1). A BLAST analysis revealed 100% sequence identity with the *CHH* mRNA of *P. clarkii* and other crustaceans. A comparison with the above mentioned GenBank sequence revealed that the sequence corresponded to the most conserved region of the *CHH* gene of the CHH family, as reported by previous in silico analysis [29]. We generated a second amplicon using isolated brain tissue, and this amplicon was also cloned and sequenced. The Clustal W alignment of the optic lobe and brain amplicons revealed a 100% sequence identity with the *CHH* mRNA sequence obtained from the eyestalk of *P. clarkii* by Yasuda-Kamatani (GenBank accession number AB027291.1). This sequence was located between exons 2 and 4 but lacked exon 3 (Figure 1, panel A). Moreover, Dircksen et al. [33] reported that this exon was necessary for the complete RNA that encodes a CHH-like peptide in the thoracic ganglion of *Carcinus maenas* but not for the *CHH* mRNA produced in the eyestalk. Subsequently, Chen et al. [28] confirmed that exon 3 was not necessary for the *P. clarkii* eyestalk *CHH* mRNA. This result suggested that the isolated RNA fragment obtained in this study does not correspond to a *CHH*-like gene but corresponds to a *CHH* RNA identical to the sequence of the eyestalk-specific CHH protein precursor, which also lacks exon 3, deposited in GenBank (AF474409) by Kuepper and Jaros. A fragment of this sequence shares 100% sequence identity with both fragments of the GenBank sequence deposited by Yasuda-Kamatani and Yasuda (2000) and the sequence obtained in this study. Briefly, both amplicons obtained in this work represent a sequence fragment corresponding to the eyestalk-specific form of CHH. Our results demonstrate that this sequence corresponds to a *CHH* mRNA, and all evidence indicates that the two pacemakers of the distributed *P. clarkii* circadian system [34], i.e., the optic lobe and brain, express the same *CHH*.

### Quantitative PCR and Circadian CHH Level Measurements

**Figure 2:**
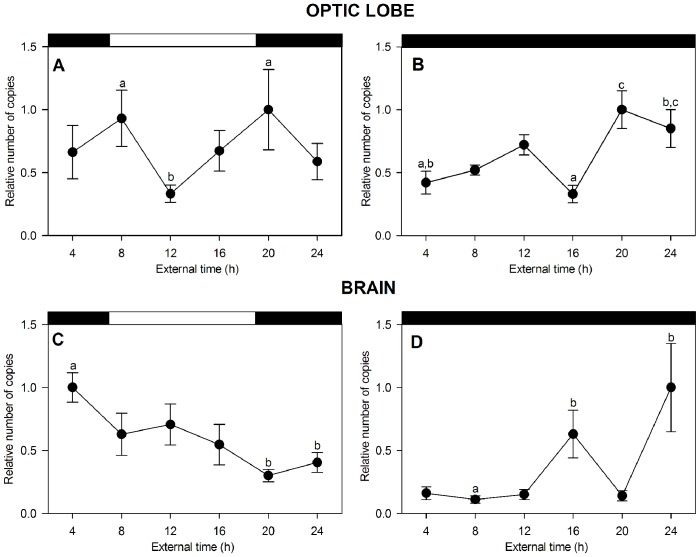
CHH mRNA levels daily and circadian oscillations in eyestalk and brain of *P. clarkii.* Chronograms showing rhythmic CHH mRNA expression in the optic lobe (A, B) and brain (C, D) under 12∶12 light–dark cycles and continuous darkness. Data are the mean ± standard error (SE) (n = 6). The results are expressed relative to the highest level of expression in each graph. The upper black and white bars in each graph denote the light and dark phases. Different letters above some time points represent statistically significant differences (post hoc analysis, LSD); please see the text for the explanation.

The temporal *CHH* expression levels were evaluated in 72 *P. clarkii* specimens collected during the spring. The animals were dissected at six time points throughout the day under both experimental conditions (LD and DD), and six animals were collected at each time point. We analyzed the *CHH* mRNA expression levels in the optic lobe and brain, which are the putative central pacemaker sites. ANOVA analysis revealed statistically significant differences between the CHH levels at different time points in both optic lobe and brain under LD (optic lobe: F = 1.395, P<0.05, LSD post hoc test 08∶00 and 20∶00 vs. 12∶00, P<0.05; brain: F = 4.7, P<0.001, LSD post hoc test 04∶00 vs. 20∶00 and 24∶00, P<0.02). Under DD, both structures showed significant differences between the time points examined (optic lobe: F = 6.5, P<0.001, LSD post hoc test 20∶00 vs. 04∶00 and 16∶00, 24∶00 vs. 16∶00, P<0.01; brain: F = 5.55, P<0.001, LSD post hoc test 24∶00 vs. 04∶00, 08∶00, 12∶00 and 20∶00, P<0.01). Chronograms showing these temporal changes in both structures are presented in Figures 2A–D. When the crayfish were subjected to an LD cycle, the optic lobe produced a bimodal rhythm (Table 2). The absolute *CHH* mRNA quantification showed that the RNA copy number increased at the end of the night and the beginning of the day (04∶00 and 08∶00, respectively), then increased at the beginning of the subjective night (20∶00) and decreased during the day (12∶00). The cosinor analysis revealed a significant bimodal rhythm with two activity peaks. The shorter peak occurred during the daytime, and this rhythm exhibited a period of 12 h (Table 2). Interestingly, under DD, the rhythmic waveform did not change, but the rhythmic parameters changed (Table 2), which showed a decrement and a phase change of 4 h, as demonstrated by the shift of the shorter peak towards the maximal peak. This phase change appears to indicate that the *CHH* levels were free-running after 4 days of darkness, demonstrating the circadian nature of this rhythm. Figure 2C shows the clear unimodal oscillation of the brain *CHH* mRNA, with the mRNA levels peaking at 04∶00. These levels abruptly decreased during the day, reaching their lowest levels at the beginning of the night (20∶00). Under the dark condition, the mRNA levels in the brain also oscillated in a circadian manner, following a bimodal waveform with an approximate 11.5-h period, as calculated by cosinor analysis. ANOVA analysis showed significant differences between the *CHH* mRNA production peaks at 16∶00 and 24∶00 (F = 5.5, P<0.001). This rhythm displayed a clear phase advance of 4 h after 4 days of darkness. The significant *CHH* level oscillations and behavior of these rhythms under LD and DD, which were apparently synchronized under LD conditions and free-running in the dark condition, prove the circadian nature of the CHH level oscillations. These findings also suggest that CHH transcription in the eyestalk and brain of *P. clarkii* is controlled by the circadian clock in a manner similar to the previously demonstrated control of CHH synthesis and secretion in the eyestalk XO-SG.

**Table 2:** Cosinor Analysis.

Parameter	LD	DD
**Eyestalk CHH**		
Period (h)	12∶00*	12∶00**
Mesor (initial # copies/rx tube)	8,230.77	16,900,000.00
Amplitude (initial # copies/rx tube)	3,321.51	7,050,000.00
Acrophase (h:min)	7∶09 hrs	10∶06 hrs
**Brain CHH**		
Period (h)	24∶00**	11∶48**
Mesor (initial # copies/rx tube)	34,800.00	10,000.00
Amplitude (initial # copies/rx tube)	15,200.00	11,100.00
Acrophase (h:min)	7∶22 hrs	6∶30 hrs

Cosinor Amplitude Significance *P*<0.05 and ** *P*<0.01.

### Different Brain Clusters and Neuropils Express CHH

**Figure 3:**
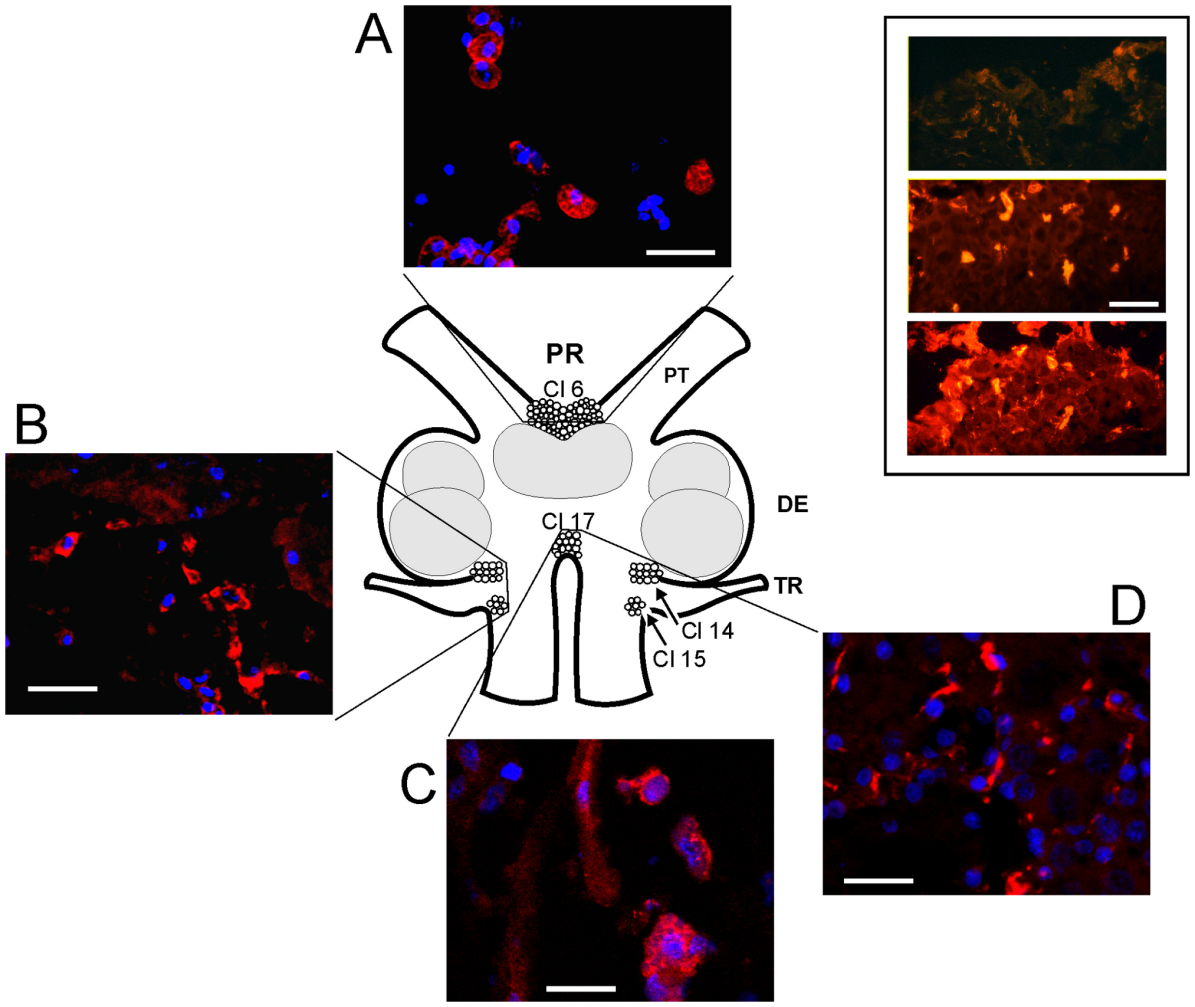
Confocal images showing brain CHH-positive cells and fibers. Immunohistochemical localization of CHH-IR cells in the crayfish brain. The drawing represents a dorsal view of the brain as they relate to the representative confocal 1.6 µm optical sections stacked in 10 µm images of CHH-IR in the neuronal clusters (Cl 6, Cl 14,15 and 17). CHH is marked in red, and the cell nucleus marked in blue (DAPI). PR, protocerebrum; DE, deuterocerebrum; TR, tritocerebrum; PT, protocerebral tract. The white calibration bars equal 40 µm. The inset shows three horizontal serial sections of PR from the dorsal (upper panel) to central (lower panel) brain (CHH is shown in red). Immunopositivity is visible in all of the sections and is brighter in the central section. White calibration bar = 100 µm.

The presence of cells that synthesize CHH in the optic lobe of *P. clarkii* has been confirmed, and the nature of these cells has been previously well characterized (for review, see [6]). To the best of our knowledge, however, this report is the first to show that *CHH* is transcribed in the brain of this species. To investigate the presence of this peptide in the brain, we used immunohistochemistry to identify the CHH-expressing cells in the brain of *P. clarkii* using an antiserum that was previously tested in the *P. clarkii* optic lobe [32], [24]. Confocal microscopy revealed CHH-IR in the fibers and cells bodies of the protocerebral and tritocerebral clusters and neuropils. A schematic representation of the various crayfish brain structures is shown in Figure 3. This figure shows representative immunostained confocal images of protocerebral (A) and tritocerebral cells (B–D) expressing CHH in the perikarya (red); the nuclei were counterstained with DAPI (blue).

**Figure 4:**
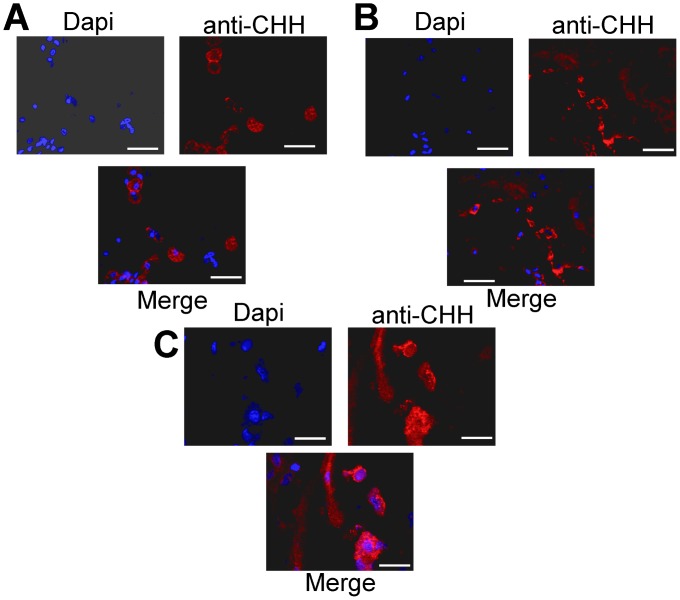
Imunohistochemical localization of CHH in perikarya and nuclei in the central brain of crayfish. Stacked confocal images of cluster 6 of the protocerebrum (A) and tritocerebral clusters 14 and 15 (B) and cluster 17 (C) of *P. clarkii* with small CHH-immunoreactive somata and some fibers (red). Nuclei are counterstained with DAPI (blue). Arrowheads show CHH-IR in the perikarya and the nuclei. Each image shows the blue (DAPI) and red (anti-CHH) channels as well as the merged image. Horizontal white bars = 40 µm.

CHH-IR was detected in the perikarya of 13 neurons of protocerebral cluster 6 (see Figure 3 A and 4A). Although this immunoreactivity appeared to circumscribe the perikarya, this observation could be an effect of the 10 µm optical section stacks. The CHH-IR also appeared to localize to the nuclei, as shown by co-localization with DAPI staining (Figure 4 C). CHH-IR was also observed in the tritocerebral antenna II neuropil and clusters 14 and 15. Figure 3 B shows CHH-IR fibers and 16 cell bodies. Figure 3C–D also shows that medial cluster 17 exhibits CHH-IR in 8 cell bodies and in a fiber that most likely projects to esophageal connections. The neurons showed strong CHH-IR in both the perikarya and nuclei, suggesting an interaction between these two cell structures. This interaction can be observed in Figures 4A–C, which shows DAPI, CHH-IR and the corresponding merged images for each structure; A) protocerebral cluster 6, B) tritocerebral clusters 14 and 15 and C) tritocerebral cluster 17. The oval cell bodies size average was 59 µm long×38 µm large ±7 µm. The biggest cells were found in cluster 6 and the smallest cells were found in clusters 14 and 15.

The presence of these cell bodies in the locus where the clock proteins PER, TIM and CLOCK have been reported (cluster 6 and TIM in cluster 17) [35] and the CHH interaction in the perikarya and nuclei suggest a relationship between the clock transcription-translation feedback loop and CHH, as has been reported for other neuropeptides [36].
